# Associations of fruit & vegetable intake and physical activity with poor self-rated health among Chinese older adults

**DOI:** 10.1186/s12877-021-02709-6

**Published:** 2022-01-03

**Authors:** Ming Guan

**Affiliations:** 1 Family Issues Center, Xuchang University, Road Bayi 88, Xuchang, Henan China; 2 International Issues Center, Xuchang University, Road Bayi 88, Xuchang, Henan China; 3 School of Business, Xuchang University, Road Bayi 88, Xuchang, Henan China

**Keywords:** Chinese older adults, Servings of F&V intake, Levels of physical activity, Poor SRH, Sociodemographic factors

## Abstract

**Background:**

Despite the existing literature highlights the central roles of sociodemographic factors, fruit & vegetable (F&V) intake, and physical activities for maintaining good health, less is known about the associations in the Chinese context. This study attempted to explore the associations of servings of F&V intake and levels of physical activities with poor self-rated health (SRH) among Chinese older adults.

**Methods:**

Data were drawn from the Study on Global Ageing and Adult Health-China (SAGE-China) issued by the World Health Organization and included 7560 respondents aged ≥60 years in China. After screening out the potential confounding factors, multiple logistic regression models were adopted to explore the associations of sociodemographic factors, servings of F&V intake, and levels of physical activities with poor SRH.

**Results:**

Among the sample, nearly a quarter reported poor health status. There were significant gender differences in the case of servings of F&V intake and levels of physical activities. Logistic regressions indicated that higher fruit intake was associated with lower likelihood of vigorous level of physical activity as compared to zero intake. Likewise, higher vegetable intake (≥10 servings) was associated with a higher likelihood of vigorous & moderate level of physical activity when compared to lower intake (≤ 4 servings). Higher fruit intake was associated with a lower likelihood of poor SRH. Similarly, vegetable intake (5 servings: AOR = 0.69, 95%CI: 0.58–0.83; 6–9 servings: AOR = 0.72, 95%CI: 0.59–0.87) was significantly associated with poor SRH. Additionally, vigorous level of physical activity (AOR = 0.79, 95%CI: 0.65–0.97) and vigorous fitness/leisure (AOR = 0.57, 95%CI: 0.39–0.84) were significantly associated with poor SRH.

**Conclusion:**

This study suggested that older adults with high fruit intake had lower probability of performing vigorous & moderate level of physical activity, while those with high vegetable intake had higher probability of performing vigorous & moderate level of physical activity. Likewise, the older adults with high F&V intake and higher probability of performing vigorous level of physical activity, walk/bike activity, and vigorous/moderate fitness/leisure had less likelihood to face the risk for poor SRH outcomes. The appropriate servings of F&V intake and levels of physical activity should be highlighted.

**Supplementary Information:**

The online version contains supplementary material available at 10.1186/s12877-021-02709-6.

## Background

There is an increased attention towards health status among the older adults in China. Many studies research found there was high prevalence of normal-weight obesity [1], cognitive impairment [2], anaemia [3], and reduced kidney function [4] among Chinese older adults. Likewise, a meta-analysis indicated estimated proportions of sleep duration < 5 h/day and < 6 h/day were 18.8% (95% CI: 1.7–35.9%) and 26.7% (95% CI: 19.7–33.7%) in Chinese older adult population, respectively [5]. Additionally, Chinese older adults demonstrated high incidences of physical inactivity (62.5%) and unhealthy dietary behavior (45.7%) [6]. Similarly, a study reported Chinese older adults aged > 70 years performed physical activities quite frequently with low and medium intensity for a short time period [7]. Moreover, a high proportion (27.71%) of older adults with poor SRH was confirmed in the Chinese individuals aged ≥60 years [8].

Since entry into the ageing society, the associated factors of general health status among Chinese older adults have attracted academic attention [9]. In China, SRH was consistent with objective health status and could serve as a global measure of health status in the general population [10]. SRH could directly predict the subjective well-being [11] and be reflected by income inequality among Chinese older adults [12]. China had some differences from other countries regarding how sociodemographic factors and behaviors influenced SRH [13–16]. Likewise, an empirical outcome indicated that associations between socioeconomic disadvantages and health changes in China were possibly similar to those found in western populations [17]. These prior studies collectively suggested that a similar research on the associations regarding SRH in the field of Chinese geriatric research was needed.

Prior research confirmed that sociodemographic factors contributed to servings of F&V intake, levels of physical activities, and poor SRH in western countries. For example, economic level and social characteristics were associated with low F&V intake within the deprived French population [18]. Simultaneously, a study reported that educational level was correlated to levels of physical activities [19]. Another study indicated that the correlations of physical activities with sociodemographic and health factors differed significantly from levels of physical activities [20]. Likewise, education [21], income [22], area factors [23], marital status [24], occupations [25], and living arrangements [26] were reported to be associated with SRH. Thus, when assessing the associations of servings of F&V intake and levels of physical activities with poor SRH, sociodemographic factors could possibly be the potential confounding factors.

There were several theories regarding the relationship between physical activities and health promotion, including health behavior theories [27], theory of planned behavior [28], multi-theory model of health behavior change [29], and health belief model [30]. The core proposition of those theories was that physical activities could promote health status among various population groups. However, scarce theoretical relationship between diet and health promotion was reported. Particularly, normalization process theory was proved to be useful in conceptualizing barriers to implementation of the nutrition guidelines for older people [31]. Additionally, theory of planned behavior was employed to predict physical activities and F&V intake of young rather than older population [32]. Here, the theories above were adopted as theoretical foundation and analytical framework for analyzing the associations of F&V intake and physical activities with poor SRH.

The relationship between servings of F&V intake and levels of physical activities was documented in the western world. For instance, a cross-sectional study in the Netherlands concluded physically active older adults tended to consume more F&V compared to less active peers [33]. Another cross-sectional study among 810 adults aged ≥85 years indicated that high levels of physical activity could be achieved by improving overall diet [34]. Based on prior research, this study could hypothesize below,

- Hypothesis 1: Controlling for potentially confounding factors, lower fruit intake has significantly negative association with higher levels of physical activities in Chinese older adults.
- Hypothesis 2: Controlling for potentially confounding factors, lower vegetable intake has significantly negative association with higher levels of physical activities in Chinese older adults.

Simultaneously, the association of servings of F&V intake with SRH has been well documented in western countries. For example, two studies in UK and Portugal showed that servings of F&V intake were associated with SRH [35, 36]. Clinically, F&V intake could improve health status of patients [37–39]. Specifically, research has shown appropriate servings of F&V intake could prevent cognitive dysfunction [40], mental disorders [41], and cardiovascular disease [42]. Thus, the following hypotheses were posited:

- Hypothesis 3: Controlling for potentially confounding factors, lower fruit intake has significantly positive association with poor SRH in Chinese older adults.
- Hypothesis 4: Controlling for potentially confounding factors, lower vegetable intake has significantly positive association with poor SRH in Chinese older adults.

Furthermore, it was confirmed scientific intensity of physical activity could benefit and improve physical and mental health [43, 44]. Likewise, a cross-sectional study reported long working hours were associated with increased risk of poor SRH [45]. Also, a cross-sectional study highlighted the potential for promotion of physical activity to reduce socioeconomic inequalities in SRH [46]. In a similar vein, several prior studies demonstrated that good SRH was supported by physical activities in adults [47–49] and adolescents [50–53]. Additionally, a review revealed the mechanisms of physical activity causing clinical changes in health status [54]. Based on previous research, the following hypothesis was given:

- Hypothesis 5: Controlling for potentially confounding factors, higher levels of physical activities have significantly negative association with poor SRH in Chinese older adults.

## Method

### Data

The publically available dataset regarding the health and physical activities of older populations in China, titled the Study on Global Ageing and Adult Health-China (SAGE-China), was used in this study. The SAGE-China survey with a multistage stratified cluster sample design was completed as a face-to-face household survey in China in 2010. In SAGE-China, there were 95% response rate for the household questionnaire and > 98% response rate for the individual questionnaire. After excluding the 7449 persons aged < 60 years and 41 missing values, 7560 Chinese older adults aged ≥60 years were obtained from the dataset.

### Main variables

#### Dependent variables

The dependent variables were poor SRH and physical activities. SRH was assessed by the question: “In general, how would you rate your health today?” with five-point Likert scales ranging from“1 =very good” to “5 = very bad”. Thus, poor SRH was obtained by dichotomizing the response options into binary values: 1 (poor = bad/very bad) and 0 (good = very good/good/so so). Physical activities (time ≥ 10 min) mainly included moderate level of physical activity, vigorous level of physical activity, walk/bike activity, moderate fitness/leisure, and vigorous fitness/leisure. The response options of physical activities were yes (=1) and no (=0).

#### Independent variables

The main independent variables were physical activities, fruit intake, and vegetable intake. Fruit intake was measured by the question: “How many servings of fruit do you eat on a typical day?” Respondents reported the number of servings they consumed banana, mango, apple, orange, papaya, tangerine, grapefruit, peach, and pear. Vegetable intake was measured by the question: “How many servings of vegetables do you eat on a typical day?” Respondents reported the number of servings they consumed tomatoes, cauliflowers, potatoes, cucumbers, peas, corns, lettuces, squashes, and beans. With respect to statistical density, respondents’ servings of fruit and vegetable intake were divided by 0, 1, 2, 3, and ≥ 4 and 0–4, 5, 6–9, 10, and ≥ 11, respectively.

### Sociodemographic factors

The sociodemographic variables included age, gender, marital status, ever schooled, ethnicity, and always lived in this village/town/city. Age was divided into two groups: 60 to 69 years and 70 years and above. Gender was categorized as female (=0) and male (=1). Marital status was categorized as never married, currently married, cohabited, separated/divorced, and widowed status. Ever schooled level was divided by no (=0) and yes (=1). Ethnicity was grouped by Han majority (=0) and ethnic minority (=1). The response options of “always lived in this village/town/city” were classified into no (=0) and yes (=1).

### Statistical analyses

First, descriptive statistics were carried out by Chi-square tests with percentages to reflect the sample characteristics. Second, principal component analysis (PCA) with varimax rotation and method of maximum likelihood was performed regarding the factor analysis of physical activities. In PCA, components with an eigenvalues > 1 were retained. In the exploratory factor analysis, component loadings < 0.5 were deleted. PCA was conducted by SPSS 22.0. Regarding convergent validity and discriminant validity assessment, squared correlations among latent variables and average variances extracted (AVEs) were calculated with Stata program “*condisc*” [55]. According to Fornell and Larcker (1981), AVE values less than 0.5 was rejected [56]. Consequently, the five domains of physical activities could separately conduct as dependent variables.

On the basis of change-in-estimate criterion (> 0.09% cutoff) [57] in a stepwise fashion, the next step was to identify potential confounding factors with Stata program “*confnd*” [58] in the associations between servings of fruit intake and levels of physical activities, between servings of vegetable intake and levels of physical activities, between servings of F&V intake and poor SRH, and between levels of physical activities and poor SRH. After screening out the potential confounding factors, multiple logistic regression models of interest were conducted to explore the associations of interest among servings of fruit intake, servings of vegetable intake, levels of physical activities, and poor SRH. Here, risks were expressed as adjusted odds ratio (AOR) with 95% confidence interval (CI).

The main statistical analyses were performed using Stata version 14.0.

## Results

In Table 1, mean age of the sample was 69.79 (Standard deviation = 7.10, range = 60 to 99) years old. 24.50% of respondents reported poor health status. There were not significant gender differences in age groups. Regarding gender, number of females (52.62%) was somewhat more than number of males (47.38%). Considering marital status, there were significant gender differences among never married, currently married, cohabited, separated/divorced, and widowed status. The top two patterns of marital status were currently married (75.48%) and widowed status (22.07%), which accounted for 97.55% of the total sample. Most of the sample was educated (65.77%) and belonged to Han majority (98.66%). The number of older adults always lived in this village/town/city and that not always lived in this village/town/city was nearly equal to each other, while the number of older adults not always lived in this village/town/city were somewhat more (50.62%). Women had higher F&V intake than men. There were significant gender differences in the case of vigorous level of physical activity, moderate level of physical activity, walk/bike activity, vigorous fitness/leisure, and moderate fitness/leisure. Most of the respondents did not perform vigorous level of physical activity, vigorous fitness/leisure, or moderate fitness/leisure. Over half of them did perform not moderate level of physical activity but walk/bike activity. There were high proportions of fruit intake (≥ 2 servings), vegetable intake (≥5 servings), moderate level of physical activity, walk/bike activity, and moderate fitness/leisure.

**Table 1:** Descriptive characteristics stratified by gender among the 7560 respondents (%)

Variables	Total	Men	Women	Chi-square	*P*-value
**Poor SRH**				29.2419	0.000***
No	75.50	37.13	38.37		
Yes	24.50	10.25	14.25		
**Age group (years)**				0.1656	0.684
60–69	52.49	25.01	27.47		
≥ 70	47.51	22.42	25.09		
**Marital status**				440.5079	0.000***
Never married	0.90	0.53	0.37		
Currently married	75.48	40.68	34.80		
Cohabited	0.17	0.08	0.09		
Separated/Divorced	1.38	0.68	0.70		
Widowed	22.07	5.48	16.59		
**Ever schooled**				537.4515	0.000***
Yes	65.77	37.51	28.25		
No	34.23	9.92	24.31		
**Ethnicity**				1.2821	0.258
Han majority	98.66	46.68	51.98		
Ethnic minority	1.34	0.71	0.63		
**Always lived in this village/town/city**				292.5727	0.000***
Yes	49.38	28.45	20.93		
No	50.62	19.02	31.59		
**Fruit intake**				17.7284	0.001***
0	23.41	12.17	11.24		
1	13.21	6.14	7.06		
2	26.44	12.42	14.02		
3	14.02	6.23	7.79		
≥ 4	22.92	10.66	12.26		
**Vegetable intake**				44.4884	0.000***
0–4	27.93	11.86	16.07		
5	24.64	11.47	13.17		
6–9	19.41	9.49	9.92		
10	19.61	9.92	9.68		
≥ 11	8.41	4.71	3.70		
**Vigorous level of physical activity**				129.0088	0.000***
Yes	11.71	7.68	4.03		
No	88.29	39.66	48.63		
**Moderate level of physical activity**				3.3725	0.066*
Yes	45.17	20.87	24.30		
No	54.83	26.51	28.31		
**Walk/bike activity**				15.4592	0.000***
Yes	66.50	32.56	33.94		
No	33.50	14.77	18.73		
**Vigorous fitness/leisure**				19.5856	0.000***
Yes	4.08	2.45	1.64		
No	95.92	44.93	50.99		
**Moderate fitness/leisure**				8.7883	0.003***
Yes	15.77	8.10	7.67		
No	84.23	39.24	44.99		

Note: ***, ** and * indicates 1, 5 and 10% significance level, respectively

Regarding factor analysis of levels of physical activities, the statistical outcomes indicated adequate sample size and significant Bartlett’s test of sphericity (Kaiser-Meyer-Olkin adequacy =0.555 and Bartlett’s sphericity test: Chi-square (10) = 1494.894, *p* < 0.001). This analysis allowed to identify two factors with eigenvalue (Kaiser’s criterion) greater than 1 (1.485 and 1.167), conjointly accounting for 53.053% of the total variance. The two factor solution could be named work activity (items: vigorous level of physical activity and moderate level of physical activity) and leisure activity (items: walk/bike activity, vigorous fitness/leisure, and moderate fitness/leisure), respectively. Their Cronbach’s alpha coefficients were 0.4198 (Factor 1) and 0.2279 (Factor 2), respectively. Likewise, squared correlation between work activity and leisure activity was 0.214. AVEs were 0.341 and 0.197 in the latent variables of work activity and leisure activity, respectively. Thus, structural equation modeling was abandoned and logistic regression could be adopted.

### Association between servings of fruit intake and levels of physical activities

Supplementary Table 1a to e reported change-in-estimates for vigorous level of physical activity, moderate level of physical activity, walk/bike activity, vigorous fitness/leisure, and moderate fitness/leisure to fruit intake with the potential confounding factors. In Table 2, higher fruit intake was associated with lower odds of performing vigorous level of physical activity. Additionally, fruit intake (1 serving: AOR = 0.81, 95%CI: 0.68–0.96; ≥ 4 servings: AOR = 0.83, 95%CI: 0.73–0.95) was associated with a lower likelihood of performing physical activity at a moderate level. Similarly, fruit intake ≥1 serving was associated with a higher likelihood of performing walk/bike activity. Moreover, higher fruit intake was associated with a lower likelihood of performing vigorous fitness and leisure activities. Additionally, higher fruit intake was associated with lower odds of performing moderate fitness and leisure activities. Accordingly, Hypothesis 1 was not confirmed partially.

**Table 2:** Adjusted odds ratios (and 95% confidence intervals) of fruit intake (servings) and physical activities, AOR(95%CI)

	Vigorous level of physical activity^a^	Moderate level of physical activity^b^	Walk/bike activity^c^	Vigorous fitness/leisure^d^	Moderate fitness/leisure^e^
**Fruit intake**					
0	1 [Reference]	1 [Reference]	1 [Reference]	1 [Reference]	1 [Reference]
1	0.40***(0.29–0.55)	0.81**(0.68–0.96)	1.24**(1.01–1.52)	0.05***(0.04–0.08)	0.25***(0.20–0.30)
2	0.52***(0.42–0.65)	1.08(0.95–1.22)	1.38***(1.17–1.63)	0.05***(0.04–0.06)	0.29***(0.26–0.34)
3	0.41***(0.31–0.56)	1.10(0.93–1.30)	1.31**(1.07–1.60)	0.04***(0.03–0.06)	0.36***(0.31–0.42)
≥ 4	0.39***(0.31–0.50)	0.83***(0.73–0.95)	1.32***(1.12–1.57)	0.06***(0.04–0.07)	0.39***(0.34–0.44)
**Age group (years)**					
60–69					1 [Reference]
≥ 70					0.42***(0.38–0.47)
**Marital status**					
Never married	1 [Reference]		1 [Reference]		
Currently married	0.32***(0.28–0.38)		1.78***(1.57–2.01)		
Cohabiting	0.44(0.09–2.10)		1.56(0.40–6.09)		
Separated/divorced	0.19***(0.09–0.38)		2.44***(1.48–4.04)		
Widowed	0.18***(0.14–0.22)		1.38***(1.18–1.61)		
**Ethnicity**					
Han majority	1 [Reference]		1 [Reference]	1 [Reference]	1 [Reference]
Ethnic minority	0.47*(0.22–1.01)		0.72(0.43–1.21)	0.10***(0.03–0.35)	0.47**(0.25–0.90)
**Always lived in this village/town/city**					
Yes		1 [Reference]			
No		0.96(0.86–1.07)			

Note: ***, ** and * indicates 1, 5 and 10% significance level, respectively

*AOR* adjusted odds ratio

^a^Age group, gender, ever schooled, always lived in this village/town/city, vegetable intake, and poor SRH were screened out as the confounding variables

^b^Age group, gender, marital status, ever schooled, ethnicity, vegetable intake, and poor SRH were screened out as the confounding variables

^c^Age group, gender, ever schooled, always lived in this village/town/city, vegetable intake, and poor SRH were screened out as the confounding variables

^d^Age group, gender, marital status, ever schooled, always lived in this village/town/city, vegetable intake, and poor SRH were screened out as the confounding variables

^e^Gender, marital status, ever schooled, always lived in this village/town/city, vegetable intake, and poor SRH were screened out as the confounding variables

### Association between servings of vegetable intake and levels of physical activities

Supplementary Table 2a to e reported change-in-estimates for vigorous level of physical activity, moderate level of physical activity, walk/bike activity, vigorous fitness/leisure, and moderate fitness/leisure to vegetable intake with the potential confounding factors. In Table 3, older adults with 10 servings and ≥ 11 servings of vegetable intake had 67% (10 servings: AOR = 1.67, 95%CI: 1.30–2.13) and 275% (≥11 servings: AOR = 3.75, 95%CI: 2.89–4.87) higher probabilities of performing vigorous level of physical activity than those without it. Older adults with high vegetable intake (≥10 servings) would more likely to perform moderate level of physical activity. Older adults with high vegetable intake (≥5 servings) and fruit intake (≥1 serving) would have higher probability of performing walk/bike activity. The significantly negative association between vigorous fitness/leisure and vegetable intake was confirmed. Furthermore, the significantly negative association between moderate fitness/leisure and vegetable intake was observed. Therefore, Hypothesis 2 was not confirmed completely.

**Table 3:** Adjusted odds ratios (and 95% confidence intervals) of vegetable intake (servings) and physical activity, AOR(95%CI)

	Vigorous level of physical activity^a^	Moderate level of physical activity^b^	Walk/bike activity^c^	Vigorous fitness/leisure^d^	Moderate fitness/leisure^e^
**Vegetable intake**					
0–4	1 [Reference]	1 [Reference]	1 [Reference]	1 [Reference]	1 [Reference]
5	1.01(0.78–1.29)	1.10(0.97–1.26)	1.57***(1.32–1.86)	0.05***(0.04–0.07)	0.30***(0.26–0.36)
6–9	1.01(0.77–1.32)	1.08(0.94–1.24)	1.38***(1.15–1.65)	0.04***(0.03–0.06)	0.32***(0.27–0.37)
10	1.67***(1.30–2.13)	1.51***(1.32–1.72)	1.98***(1.65–2.37)	0.05***(0.04–0.07)	0.30***(0.25–0.35)
≥ 11	3.75***(2.89–4.87)	3.14***(2.57–3.84)	1.96***(1.54–2.49)	0.01***(0.01–0.03)	0.15***(0.12–0.20)
**Age group (years)**					
60–69					1 [Reference]
≥ 70					0.42***(0.37–0.47)
**Marital status**					
Never married	1 [Reference]		1 [Reference]		
Currently married	0.13***(0.11–0.15)		1.19*(1.00–1.42)		
Cohabiting	0.15**(0.02–0.90)		1.03(0.27–3.98)		
Separated/divorced	0.08***(0.04–0.17)		1.76**(1.05–2.96)		
Widowed	0.08***(0.06–0.10)		0.98(0.79–1.20)		
**Ever schooled**					
No			1 [Reference]		
Yes			0.95(0.83–1.08)		
**Ethnicity**					
Han majority	1 [Reference]	1 [Reference]	1 [Reference]	1 [Reference]	1 [Reference]
Ethnic minority	0.56(0.26–1.22)	0.55**(0.33–0.92)	0.70(0.40–1.20)	0.11***(0.03–0.38)	0.56(0.26–1.22)
**Always lived in this village/town/city**					
No		1 [Reference]	1 [Reference]		
Yes		0.74***(0.67–0.82)	0.96(0.85–1.09)		
**Fruit intake**					
0			1 [Reference]		
1			1.41***(1.15–1.74)		
2			1.53***(1.29–1.82)		
3			1.33***(1.08–1.63)		
≥ 4			1.25**(1.04–1.50)		
**Poor SRH**					
No		1 [Reference]			
Yes		1.01(0.89–1.15)			

Note: ***, ** and * indicates 1, 5 and 10% significance level, respectively

*AOR* adjusted odds ratio

^a^Age group, gender, ever schooled, always lived in this village/town/city, fruit intake, and poor SRH were screened out as the confounding variables

^b^Age group, gender, marital status, ever schooled, and fruit intake were screened out as the confounding variables

^c^Age group, gender, and poor SRH were screened out as the confounding variables

^d^Age group, gender, marital status, ever schooled, always lived in this village/town/city, fruit intake, and poor SRH were screened out as the confounding variables

^e^Gender, marital status, ever schooled, always lived in this village/town/city, fruit intake, and poor SRH were screened out as the confounding variables

### Association between servings of F&V intake and poor SRH

Supplementary Table 3a and b reported change-in-estimates for poor SRH to F&V intake with potential confounding factors. In Table 4, high fruit intake (≥1 serving), vigorous fitness/leisure, and walk/bike activity might be important protective factors against poor SRH. The odds ratio of poor SRH significantly associating with vegetable intake was less than 1, which indicated that higher vegetable intake could reduce the risk of poor SRH. Thus, Hypotheses 3 and 4 were confirmed completely.

**Table 4:** Adjusted odds ratios (and 95% confidence intervals) of F &V intake and poor SRH, AOR (95%CI)

	Model 1^a^	Model 2^b^
**Fruit intake**		
0	1 [Reference]	
1	0.69***(0.57–0.83)	
2	0.49***(0.41–0.57)	
3	0.33***(0.27–0.42)	
≥ 4	0.32***(0.27–0.38)	
**Vegetable intake**		
0–4		1 [Reference]
5		0.69***(0.58–0.83)
6–9		0.72***(0.59–0.87)
10		0.88(0.73–1.06)
≥ 11		1.02(0.81–1.29)
**Marital status**		
Never married		1 [Reference]
Currently married		0.39***(0.34–0.45)
Cohabiting		0.50 (0.12–2.17)
Separated/divorced		0.38***(0.22–0.64)
Widowed		0.56***(0.47–0.66)
**Ethnicity**		
Han majority	1 [Reference]	1 [Reference]
Ethnic minority	0.69(0.40–1.20)	0.84(0.50–1.43)
**Always lived in this village/town/city**		
No	1 [Reference]	1 [Reference]
Yes	0.93(0.82–1.05)	0.88*(0.77–1.00)
**Vigorous fitness/leisure**		
No	1 [Reference]	1 [Reference]
Yes	0.64**(0.43–0.94)	0.60***(0.41–0.87)
**Walk/bike activity**		
No	1 [Reference]	
Yes	0.50***(0.45–0.56)	

Note: ***, ** and * indicates 1, 5 and 10% significance level, respectively

*AOR* adjusted odds ratio

^a^Age group, gender, marital status, ever schooled, vegetable intake, vigorous level of physical activity, moderate level of physical activity, and moderate fitness/leisure were screened out as the confounding variables

^b^Fruit intake, Age group, gender, ever schooled, vigorous level of physical activity, moderate level of physical activity, walk/bike activity, and moderate fitness/leisure were screened out as the confounding variables

### Association between levels of physical activities and poor SRH

Supplementary Table 4a to e reported change-in-estimates for poor SRH to vigorous level of physical activity, moderate level of physical activity, walk/bike activity, vigorous fitness/leisure, and moderate fitness/leisure with potential confounding factors. In Table 5, vigorous level of physical activity was significantly associated with poor SRH (AOR = 0.79, 95%CI: 0.65–0.97). Older adults with walk/bike activity and high fruit intake would face less risk of poor SRH than those without. A significant association was also observed between vigorous fitness/leisure and poor SRH (AOR = 0.57, 95%CI: 0.39–0.84). Older adults with moderate fitness/leisure had a 0.31 times higher risk of poor SRH (AOR =0.31, 95%CI: 0.26–0.38) than those without it. In sum, these results provided support for Hypothesis 5.

**Table 5:** Adjusted odds ratios (and 95% confidence intervals) of physical activity and poor SRH, AOR(95%CI)

	Model 1^a^	Model 2^b^	Model 3^c^	Model 4^d^	Model 5^e^
**Vigorous level of physical activity**					
No	1 [Reference]				
Yes	0.79**(0.65–0.97)				
**Moderate level of physical activity**					
No		1 [Reference]			
Yes		1.04(0.92–1.18)			
**Walk/bike activity**					
No			1 [Reference]		
Yes			0.53***(0.46–0.60)		
**Vigorous fitness/leisure**					
No				1 [Reference]	
Yes				0.57***(0.39–0.84)	
**Moderate fitness/leisure**					
No					1 [Reference]
Yes					0.31***(0.26–0.38)
**Marital status**					
Never married	1 [Reference]	1 [Reference]	1 [Reference]	1 [Reference]	
Currently married	0.31***(0.29–0.34)	0.31***(0.28–0.35)	0.84**(0.72–0.98)	0.31***(0.29–0.33)	
Cohabiting	0.40(0.10–1.67)	0.41(0.10–1.78)	1.16(0.25–5.30)	0.38(0.09–1.62)	
Separated/divorced	0.29***(0.18–0.48)	0.30***(0.18–0.49)	0.74(0.43–1.25)	0.30***(0.18–0.50)	
Widowed	0.46***(0.40–0.52)	0.47***(0.40–0.55)	1.08(0.90–1.29)	0.46***(0.40–0.52)	
**Ethnicity**					
Han majority	1 [Reference]	1 [Reference]	1 [Reference]	1 [Reference]	1 [Reference]
Ethnic minority	0.95(0.59–1.53)	1.03(0.64–1.66)	0.69(0.40–1.19)	0.95(0.59–1.54)	0.69(0.42–1.15)
**Always lived in this village/town/city**					
No		1 [Reference]			1 [Reference]
Yes		0.87** (0.76–0.98)			0.37***(0.34–0.41)
**Fruit intake**					
0			1 [Reference]		
1			0.71***(0.57–0.88)		
2			0.50***(0.42–0.60)		
3			0.35***(0.27–0.44)		
≥ 4			0.33***(0.27–0.40)		

Note: *** and ** indicates 1 and 5% significance level, respectively

*AOR* adjusted odds ratio

^a^Fruit intake, Age group, gender, ever schooled, always lived in this village/town/city, and vegetable intake were screened out as the confounding variables

^b^Fruit intake, Age group, gender, ever schooled, and vegetable intake were screened out as the confounding variables

^c^Age group, gender, ever schooled, always lived in this village/town/city, and vegetable intake were screened out as the confounding variables

^d^Fruit intake, Age group, gender, ever schooled, always lived in this village/town/city, and vegetable intake were screened out as the confounding variables

^e^Fruit intake, Age group, gender, marital status, ever schooled, and vegetable intake were screened out as the confounding variables

## Discussion

In this cross-sectional analysis, the sample was dominated by age 60 to 69 years, females, currently married status, educated level, Han majority, good health, physical inactivity, and high F&V intake. The percentage of the respondents reported their good health status was higher than that in another study among Shanghai older adults (nearly 40%) [59]. The empirical analyses showed that older adults with higher fruit intake had lower likelihood to perform vigorous/moderate level of physical activity, higher likelihood of performing walk/bike activity, and lower likelihood of performing vigorous/moderate fitness and leisure activities. Similarly, older adults with higher vegetable intake were more likely to perform vigorous/moderate level of physical activity and walk/bike activity and less likely to perform vigorous/moderate fitness and leisure activities. Simultaneously, high F&V intake, vigorous level of physical activity, walk/bike activity, and vigorous/moderate fitness/leisure could reduce the risk of poor SRH. Therefore, the results provided useful insights into the role of servings of F&V intake and levels of physical activities in the health evaluations among the Chinese older adults.

The servings of F&V intake in this study appeared to be high because > 60% of the participants consumed ≥2 servings of fruit and > 70% consumed ≥5 servings of vegetables on an average day. Methodologically, outcome measures (≥ 2 servings of fruit and 5 servings of vegetables per day) in this study were the same with an observational study in Australia [60], which were higher than national recommendations (fruit: ≥ 2 servings/day and vegetables: ≥ 3 servings/day) in African-American adults [61]. Practically, intake servings of this sample were more appropriate than those in an Australian sample which documented 32% of adults consumed ≥2 servings of fruit and 30% consumed ≥5 servings of vegetables as recommended by the Australian Guide to Healthy Eating [62].

Regarding associations between F&V intake and various physical activities, the research outcome in this study was corroborated by the findings that quantitative servings of F&V intake and various intensities of physical activity contributed to the health status of old age. For example, a study suggested that maintaining a healthy dietary pattern and outdoor exercises was associated with a low risk of exhibiting cognitive impairment among Chinese old adults [63]. The findings in this study was also congruent with two Chinese studies in which both socioeconomic factors [64] and physical exercise [65] were related with good SRH and a Spanish investigation showing that F&V intake and physical activity could better SRH perception [66]. Similarly, another study indicated that diet quality and physical activity were known to influence health status [67].

Consistent with a number of early studies among older adults, this study documented the beneficial health effects of quantitative servings of F&V intake. For example, a study among Chinese older adults in Hong Kong concluded having ≥3 servings of vegetables and 2 servings of fruits daily might help prevent dementia [68]. A cross-sectional study in Tanzania found F&V intake (< 5 servings/day) was also associated with higher serum total cholesterol [69]. A study in Malaysia indicated inadequate F&V intake (< 5 servings/day) were all associated with physical inactivity [70]. Additionally, persons with frequent fruit consumers (≥ 4 servings/day) had a low risk of incident metabolic syndrome in a Korean sample [71]. Another study in Southern Brazil indicated that low F&V intake (≤ 4 servings/day) was defined as an indicator of unhealthy dietary habits among older adults aged > 60 years [72].

With respect to the role of sociodemographic factors in this study, several interesting results need explanations. Different from two prior studies in China [73, 74], this study convinced the role of age, ethnicity and marital status as key associating factors of the F&V intake, physical activities, and poor SRH among the Chinese older adults. In fact, age ≥ 70 years had significantly negative associations with moderate fitness/leisure. This might be explained that older adults aged ≥70 years tended to report worse health than the persons aged < 70 years. Computationally, there was turning point age for SRH was at the age of 83.69 for the SRH trend on the basis of the Chinese Longitudinal Healthy Longevity Survey [75]. Additionally, ethnic minority had significantly negative associations with physical activity. Several studies in western countries also highlighted the ethnic gradient in SRH [76, 77]. Similarly, in this study, older adults with ethnic minority were possibly characterized by physical activities. Simultaneously, marital status had significantly negative associations with vigorous level of physical activity and poor SRH and significantly positive associations with walk/bike activity. This could be partially explained by a study suggested that improving marital quality could be protective against functional abilities for older people [78].

Regarding physical activities associating F&V intake, this study was consistent with the early results. For example, a cross-sectional study identified a range of sociodemographic and behavioural factors associated with F&V intake among Chinese adults [79]. Another study convinced that physical activity and nutrition appeared to facilitate rather than hinder each other [80]. Likewise, an early study reported that F&V intake might be too specific to represent an individual’s overall nutritional status [81]. Similarly, a study in Brazil also reported the association between physical activity and F&V intake [82]. Thus, the older adults with high F&V intake might be physically active due to energy balance and healthy diet.

The benefits of physical activities and F&V intake in the current study could be explained by a number of early studies. For example, a cross-sectional survey showed that combination of sufficient F&V intake and adequate physical activity was significantly associated with reduced metabolic syndrome risk among adult residents of China [83]. Simultaneously, higher levels of physical activities and daily F&V intake might be protective against cognitive decline in older adults [84]. Furthermore, a study among community-dwelling older adults indicated F&V intake could reduce a low short-term risk of frailty [85]. Another study suggested that a modest increase in F&V intake or leisure-time physical activity could remarkably influence SRH of older adults [86].

Considering F&V intake associating health status, this study was consistent with the early findings. For instance, higher F&V intake was associated with a lower risk of frailty in this cohort of US women aged ≥60 years [87] and a reduced risk of total mortality for Chinese adults [88]. In this study, higher F&V intake was related to a lower likelihood of poor SRH. This might be explained by several early studies. For example, multiple studies indicated that frequent intake of F&V was associated with the perception of better health [89, 90] and could reduce cardiovascular disease mortality [91] and blood triglyceride levels [92].

Regarding the physical activities associating poor SRH, this study was in line with previous studies. For example, several studies indicated physical activities were important predictors for SRH [93, 94]. In China, physical inactivity contributed 12–19% to the risks associated with five major non-communicable diseases [95]. Similarly, another cross-sectional study concluded poor SRH status was related to a low likelihood of physical activity [96]. Likewise, another cross-sectional study concluded physical activity was a significant protective factor against metabolic syndrome in older women [97].

Furthermore, the relationship between various forms of physical activity and poor SRH could be explained by a series of health literature. For example, a mail-in survey study concluded moderate physical activity could lead to adoption of other healthy behaviors [98]. A cross-sectional study suggested that intensity of physical activity was an important contributor to neuronal function [99]. Similarly, a study in young adults suggested that vigorous level of physical activity was more closely associated with high heart rate complexity than moderate level of physical activity [100]. Additionally, multiple findings suggested that leisure-time physical activity was associated with better SRH [101, 102].

Notably, unhealthy eating habits are risk factors for poor health status among Chinese older adults. High salt intake is one of poor eating patterns. The sodium eating pattern is mainly characterized by pickled or salted vegetables, salted seafood, salted nuts and potato, and bacon in China. Moreover, salt added during cooking is preferred by common Chinese persons. Thus, the national average cooking salt intake exceeded recommendations of the World Health Organization (< 5 g/day of salt) [103] and Chinese proposed intake for preventing non-communicable chronic diseases [104]. Currently, high dietary sodium intake is so difficult to control by individuals that only governmental intervention can reduce dietary sodium intake and subsequent blood pressure [105].

### Strength, limitations, and future directions

There were three main strengths in this study. First, based on serving’s density, fruit and vegetable intake were categorized in this study, respectively. Second, identifying and screening out confounding variables in this study was on the basis of change-in-estimate criterion. Third, the findings of tentative analysis (component loadings > 0.5, AVE values < 0.5, and Cronbach’s alpha coefficients < 0.5) led to abandonment of structural equation model design.

This study had three main limitations. First, based on a survey, the assessments were all questionnaire-based and often referring only to single items, which might limit the number of the variables of interest. Besides, surveyed data of levels of physical activities and servings of F&V intake were less inaccurate than clinical diagnose data. Simultaneously, the findings here could not reflect the causal relationships among the three variables of interest. Second, the rough assessment of physical activities (10 min criteria) rather than World Health Organization recommendations for physical activity in older adults (≥ 150 min of moderate-intensity aerobic physical activity (APA) or ≥ 75 min of vigorous-intensity APA throughout the week) was an obvious limitation of this study. Third, information about cognitive and functional status which was often considered as two important potential confounding factors was not analyzed in the present study.

Some future directions could be derived from this study. First, further studies could reanalyze the associations of interest on the basis of large sample and longitudinal data. Second, discrepancies between early outcomes in the western world and the findings in the present study could be studied further with the relevant survey datasets. Finally, mediating and moderating effects of servings of F&V intake and levels of physical activities on poor SRH might be further explored.

### Practical implications

F&V intake and physical activities are the key elements of healthy ageing and important factors of modifiable risk in the prevention of poor SRH. There are several implications for health practitioners from the findings in this study. First, it was feasible to adopt servings of F&V intake and levels of physical activities to rate and assess subjective health status. Second, health policy-designers could redesign quantities of F&V intake and exercise-friendly neighborhoods in order to promote health status among Chinese older adults. Third, in order to lead a meaningful life of successful ageing, Chinese older adults should be aware of health benefits of F&V intake and physical activities and harmful impacts of servings, intensity, and frequency. Thus, the health policymakers should address both servings of F&V intake and levels of physical activities in cost-effective ways towards healthy Chinese older adults.

## Conclusion

In conclusion, high F&V intake, vigorous level of physical activity, walk/bike activity, and vigorous/moderate fitness/leisure were protective factors against poor SRH among Chinese older adults. As the protective lifestyle behaviors, appropriate servings of F&V intake and levels of physical activities according to individual health status should be educated, arranged, and implemented among Chinese older adults. Globally, the empirical outcomes in this study could be generalized as references for other countries. Future research is needed to initiate panel studies to address causality in the relationships among F&V intake, physical activities, and poor SRH among Chinese older adults.

## Supplementary Information

**Additional file 1: Supplementary Table 1.** a. Change-in-estimate for vigorous level of physical activity to fruit intake with possible confounding factors (*n* = 6770). b. Change-in-estimate for moderate level of physical activity to fruit intake with possible confounding factors (*n* = 6760). c. Change-in-estimate for walk/bike activity to fruit intake with possible confounding factors (*n* = 6755). d. Change-in-estimate for vigorous fitness/leisure to fruit intake with possible confounding factors (*n* = 6754). e. Change-in-estimate for moderate fitness/leisure to fruit intake with possible confounding factors (*n* = 6753). **Supplementary Table 2.** a. Change-in-estimate for vigorous level of physical activity to vegetable intake with possible confounding factors (*n* = 6770). b. Change-in-estimate for moderate level of physical activity to vegetable intake with possible confounding factors (*n* = 6760). c. Change-in-estimate for walk/bike activity to vegetable intake with possible confounding factors (*n* = 6755). d. Change-in-estimate for vigorous fitness/leisure to vegetable intake with possible confounding factors (*n* = 6754). e. Change-in-estimate for moderate fitness/leisure to vegetable intake with possible confounding factors (*n* = 6753). **Supplementary Table 3.** a. Change-in-estimate for poor SRH to fruit intake with possible confounding factors (*n* = 6695). b. Change-in-estimate for poor SRH to vegetable intake with possible confounding factors (*n* = 6695). **Supplementary Table 4.** a. Change-in-estimate for poor SRH to vigorous level of physical activity with possible confounding factors (*n* = 6770). b. Change-in-estimate for poor SRH to moderate level of physical activity with possible confounding factors (*n* = 6760). c. Change-in-estimate for poor SRH to walk/bike activity with possible confounding factors (*n* = 6755). d. Change-in-estimate for poor SRH to vigorous fitness/leisure with possible confounding factors (*n* = 6754). e. Change-in-estimate for poor SRH to moderate fitness/leisure with possible confounding factors (*n* = 6753).

## Data Availability

The datasets analysed during the current study were available in the WHO Multi-Country Studies Data Archive, https://apps.who.int/healthinfo/systems/surveydata/index.php/catalog/141.

## Abbreviations

F&V: Fruit & vegetable
SRH: Self-rated health
SAGE-China: Study on Global Ageing and Adult Health-China
DSPs: Death Surveillance Points
PCA: Principal component analysis
AVE: Average variances extracted
AOR: Adjusted odds ratio
APA: Aerobic physical activity
