# Supplementary material for: Associations of fruit & vegetable intake and physical activity with poor self-rated health among Chinese older adults
Source: BMC Geriatr. 2022 Jan 3;22:10. doi: 10.1186/s12877-021-02709-6 (PMC8722069; doi:10.1186/s12877-021-02709-6)
Supplement: Supplementary file 1 — Additional file 1: Supplementary Table 1. a. Change-in-estimate for vigorous level of physical activity to fruit intake with possible confounding factors (n = 6770). b. Change-in-estimate for moderate level of physical activity to fruit intake with possible confounding factors (n = 6760). c. Change-in-estimate for walk/bike activity to fruit intake with possible confounding factors (n = 6755). d. Change-in-estimate for vigorous fitness/leisure to fruit intake with possible confounding factors (n = 6754). e. Change-in-estimate for moderate fitness/leisure to fruit intake with possible confounding factors (n = 6753). Supplementary Table 2. a. Change-in-estimate for vigorous level of physical activity to vegetable intake with possible confounding factors (n = 6770). b. Change-in-estimate for moderate level of physical activity to vegetable intake with possible confounding factors (n = 6760). c. Change-in-estimate for walk/bike activity to vegetable intake with possible confounding factors (n = 6755). d. Change-in-estimate for vigorous fitness/leisure to vegetable intake with possible confounding factors (n = 6754). e. Change-in-estimate for moderate fitness/leisure to vegetable intake with possible confounding factors (n = 6753). Supplementary Table 3. a. Change-in-estimate for poor SRH to fruit intake with possible confounding factors (n = 6695). b. Change-in-estimate for poor SRH to vegetable intake with possible confounding factors (n = 6695). Supplementary Table 4. a. Change-in-estimate for poor SRH to vigorous level of physical activity with possible confounding factors (n = 6770). b. Change-in-estimate for poor SRH to moderate level of physical activity with possible confounding factors (n = 6760). c. Change-in-estimate for poor SRH to walk/bike activity with possible confounding factors (n = 6755). d. Change-in-estimate for poor SRH to vigorous fitness/leisure with possible confounding factors (n = 6754). e. Change-in-estimate for poor SRH to moderate fitness/le [file 12877_2021_2709_MOESM1_ESM.docx]

**Supplementary Table 1**

Supplementary table 1a. Change-in-estimate for vigorous level of physical activity to fruit intake with possible confounding factors (n= 6770).

| Variables removed | Odds ratio | 95% lower limit | 95% upper limit | Change, % |
| --- | --- | --- | --- | --- |
| Adj.All | 0.8787 | 0.8337 | 0.9261 |  |
| **-marital status** | **0.8791** | **0.8342** | **0.9265** | **0.0559** |
| **-ethnicity** | **0.8797** | **0.8348** | **0.9271** | **0.0656** |
| -age | 0.8715 | 0.8272 | 0.9181 | 0.9370 |
| -ever schooled | 0.8614 | 0.8182 | 0.9068 | 1.1564 |
| -gender | 0.8539 | 0.8114 | 0.8987 | 0.8690 |
| -poor SRH | 0.8677 | 0.8252 | 0.9124 | 1.6162 |
| -vegetable intake | 0.8812 | 0.8370 | 0.9277 | 1.5503 |
| - always lived in this village/town/city | 0.8579 | 0.8152 | 0.9028 | 2.6417 |

Supplementary table 1b. Change-in-estimate for moderate level of physical activity to fruit intake with possible confounding factors (n= 6760) .

| Variables removed | Odds ratio | 95% lower limit | 95% upper limit | Change, % |
| --- | --- | --- | --- | --- |
| Adj.All | 0.8779 | 0.8470 | 0.9100 |  |
| **- always lived in this village/town/city** | **0.8775** | **0.8467** | **0.9094** | **0.0515** |
| -ethnicity | 0.8784 | 0.8476 | 0.9103 | 0.1073 |
| -marital status | 0.8773 | 0.8465 | 0.9091 | 0.1297 |
| -poor SRH | 0.8787 | 0.8482 | 0.9103 | 0.1620 |
| -gender | 0.8806 | 0.8502 | 0.9120 | 0.2122 |
| -age | 0.8766 | 0.8468 | 0.9075 | 0.4453 |
| -ever schooled | 0.8592 | 0.8306 | 0.8889 | 1.9839 |
| -vegetable intake | 0.8897 | 0.8608 | 0.9195 | 3.5408 |

Supplementary table 1c. Change-in-estimate for walk/bike activity to fruit intake with possible confounding factors (n= 6755) .

| Variables removed | Odds ratio | 95% lower limit | 95% upper limit | Change, % |
| --- | --- | --- | --- | --- |
| Adj.All | 0.9964 | 0.9595 | 1.0347 |  |
| **-marital status** | **0.9970** | **0.9601** | **1.0353** | **0.0622** |
| **-ethnicity** | **0.9977** | **0.9608** | **1.0361** | **0.0739** |
| -ever schooled | 0.9954 | 0.9592 | 1.0330 | 0.2311 |
| -gender | 0.9936 | 0.9575 | 1.0311 | 0.1825 |
| -age | 0.9956 | 0.9597 | 1.0329 | 0.2021 |
| - always lived in this village/town/city | 0.9985 | 0.9627 | 1.0356 | 0.2850 |
| -vegetable intake | 1.0140 | 0.9783 | 1.0509 | 1.5536 |
| -poor SRH | 1.0438 | 1.0078 | 1.0810 | 2.9384 |

Supplementary table 1d. Change-in-estimate for vigorous fitness/leisure to fruit intake with possible confounding factors (n= 6754) .

| Variables removed | Odds ratio | 95% lower limit | 95% upper limit | Change, % |
| --- | --- | --- | --- | --- |
| Adj.All | 1.1081 | 1.0155 | 1.2090 |  |
| **-ethnicity** | **1.1091** | **1.0165** | **1.2101** | **0.0918** |
| -marital status | 1.1105 | 1.0180 | 1.2116 | 0.1310 |
| -age | 1.1068 | 1.0145 | 1.2075 | 0.3354 |
| - always lived in this village/town/city | 1.1179 | 1.0252 | 1.2190 | 1.0020 |
| -vegetable intake | 1.1016 | 1.0126 | 1.1983 | 1.4614 |
| -gender | 1.0856 | 0.9981 | 1.1807 | 1.4542 |
| -poor SRH | 1.1032 | 1.0152 | 1.1989 | 1.6271 |
| -ever schooled | 1.1344 | 1.0454 | 1.2310 | 2.8249 |

Supplementary table 1e. Change-in-estimate for moderate fitness/leisure to fruit intake with possible confounding factors (n=6753) .

| Variables removed | Odds ratio | 95% lower limit | 95% upper limit | Change, % |
| --- | --- | --- | --- | --- |
| Adj.All | 1.1755 | 1.1204 | 1.2332 |  |
| **-age** | **1.1757** | **1.1207** | **1.2334** | **0.0193** |
| **-ethnicity** | **1.1766** | **1.1216** | **1.2343** | **0.0785** |
| -marital status | 1.1744 | 1.1195 | 1.2319 | 0.1914 |
| -gender | 1.1684 | 1.1140 | 1.2254 | 0.5113 |
| -vegetable intake | 1.1826 | 1.1278 | 1.2401 | 1.2174 |
| -poor SRH | 1.2026 | 1.1474 | 1.2605 | 1.6953 |
| - always lived in this village/town/city | 1.2259 | 1.1703 | 1.2841 | 1.9319 |
| -ever schooled | 1.2644 | 1.2080 | 1.3234 | 3.1440 |

**Supplementary Table 2**

Supplementary table 2a. Change-in-estimate for vigorous level of physical activity to vegetable intake with possible confounding factors (n=6770).

| Variables removed | Odds ratio | 95% lower limit | 95% upper limit | Change, % |
| --- | --- | --- | --- | --- |
| Adj.All | 1.3583 | 1.2828 | 1.4381 |  |
| **-marital status** | **1.3586** | **1.2831** | **1.4384** | **0.0230** |
| **-ethnicity** | **1.3591** | **1.2836** | **1.4390** | **0.0381** |
| -ever schooled | 1.3539 | 1.2789 | 1.4333 | 0.3783 |
| -poor SRH | 1.3498 | 1.2752 | 1.4287 | 0.3073 |
| -fruit intake | 1.3408 | 1.2663 | 1.4196 | 0.6671 |
| - always lived in this village/town/city | 1.3500 | 1.2748 | 1.4295 | 0.6848 |
| -gender | 1.3747 | 1.2990 | 1.4549 | 1.8354 |
| -age | 1.4154 | 1.3380 | 1.4973 | 2.9579 |

Supplementary table 2b. Change-in-estimate for moderate level of physical activity to vegetable intake with possible confounding factors (n=6760).

| Variables removed | Odds ratio | 95% lower limit | 95% upper limit | Change, % |
| --- | --- | --- | --- | --- |
| Adj.All | 1.3500 | 1.2979 | 1.4042 |  |
| **- always lived in this village/town/city** | **1.3503** | **1.2982** | **1.4045** | **0.0224** |
| **-poor SRH** | **1.3499** | **1.2978** | **1.4040** | **0.0319** |
| **-ethnicity** | **1.3505** | **1.2984** | **1.4046** | **0.0467** |
| -marital status | 1.3491 | 1.2971 | 1.4031 | 0.1051 |
| -gender | 1.3468 | 1.2952 | 1.4006 | 0.1650 |
| -ever schooled | 1.3336 | 1.2829 | 1.3863 | 0.9831 |
| -age | 1.3593 | 1.3082 | 1.4124 | 1.9279 |
| -fruit intake | 1.3297 | 1.2806 | 1.3807 | 2.1805 |

Supplementary table 2c. Change-in-estimate for walk/bike activity to vegetable intake with possible confounding factors (n=6755) .

| Variables removed | Odds ratio | 95% lower limit | 95% upper limit | Change, % |
| --- | --- | --- | --- | --- |
| Adj.All | 1.1379 | 1.0920 | 1.1857 |  |
| **-ever schooled** | **1.1377** | **1.0918** | **1.1855** | **0.0157** |
| **-ethnicity** | **1.1381** | **1.0923** | **1.1860** | **0.0365** |
| **-marital status** | **1.1387** | **1.0928** | **1.1865** | **0.0481** |
| **-fruit intake** | **1.1380** | **1.0925** | **1.1853** | **0.0631** |
| **- always lived in this village/town/city** | **1.1370** | **1.0915** | **1.1843** | **0.0883** |
| -poor SRH | 1.1358 | 1.0906 | 1.1829 | 0.1004 |
| -gender | 1.1414 | 1.0961 | 1.1886 | 0.4936 |
| -age | 1.1651 | 1.1194 | 1.2126 | 2.0719 |

Supplementary table 2d. Change-in-estimate for vigorous fitness/leisure to vegetable intake with possible confounding factors (n=6754) .

| Variables removed | Odds ratio | 95% lower limit | 95% upper limit | Change, % |
| --- | --- | --- | --- | --- |
| Adj.All | 0.8915 | 0.8097 | 0.9816 |  |
| **-ethnicity** | **0.8917** | **0.8099** | **0.9819** | **0.0262** |
| -marital status | 0.8926 | 0.8108 | 0.9828 | 0.1041 |
| -ever schooled | 0.8939 | 0.8119 | 0.9841 | 0.1392 |
| -poor SRH | 0.8923 | 0.8107 | 0.9822 | 0.1729 |
| - always lived in this village/town/city | 0.8896 | 0.8087 | 0.9785 | 0.3095 |
| -age | 0.8998 | 0.8183 | 0.9894 | 1.1492 |
| -gender | 0.9146 | 0.8321 | 1.0052 | 1.6409 |
| -fruit intake | 0.9359 | 0.8540 | 1.0257 | 2.3367 |

Supplementary table 2e. Change-in-estimate for moderate fitness/leisure to vegetable intake with possible confounding factors (n=6753) .

| Variables removed | Odds ratio | 95% lower limit | 95% upper limit | Change, % |
| --- | --- | --- | --- | --- |
| Adj.All | 1.0873 | 1.0319 | 1.1457 |  |
| **-ethnicity** | **1.0877** | **1.0322** | **1.1461** | **0.0320** |
| **-age** | **1.0872** | **1.0320** | **1.1453** | **0.0476** |
| -poor SRH | 1.0846 | 1.0298 | 1.1423 | 0.2369 |
| -marital status | 1.0818 | 1.0273 | 1.1393 | 0.2563 |
| -gender | 1.0855 | 1.0309 | 1.1430 | 0.3386 |
| -ever schooled | 1.0956 | 1.0407 | 1.1533 | 0.9310 |
| - always lived in this village/town/city | 1.0816 | 1.0283 | 1.1378 | 1.2725 |
| -fruit intake | 1.1145 | 1.0614 | 1.1703 | 3.0428 |

**Supplementary Table 3**

Supplementary table 3a. Change-in-estimate for poor SRH to fruit intake with possible confounding factors (n= 6695) .

| Variables removed | Odds ratio | 95% lower limit | 95% upper limit | Change, % |
| --- | --- | --- | --- | --- |
| Adj.All | 0.7975 | 0.7648 | 0.8315 |  |
| **-ethnicity** | **0.7974** | **0.7648** | **0.8314** | **0.0068** |
| **- always lived in this village/town/city** | **0.7971** | **0.7646** | **0.8310** | **0.0328** |
| **-vigorous fitness/leisure** | **0.7967** | **0.7642** | **0.8306** | **0.0556** |
| -marital status | 0.7957 | 0.7633 | 0.8295 | 0.1249 |
| -age | 0.7966 | 0.7642 | 0.8304 | 0.1128 |
| -vigorous level of physical activity | 0.7986 | 0.7662 | 0.8324 | 0.2507 |
| -vegetable intake | 0.8008 | 0.7683 | 0.8346 | 0.2776 |
| -moderate level of physical activity | 0.7981 | 0.7658 | 0.8317 | 0.3434 |
| **-walk/bike activity** | **0.7984** | **0.7663** | **0.8318** | **0.0388** |
| -gender | 0.8031 | 0.7710 | 0.8365 | 0.5890 |
| -moderate fitness/leisure | 0.7939 | 0.7624 | 0.8268 | 1.1405 |
| -ever schooled | 0.7661 | 0.7362 | 0.7971 | 3.5087 |

Supplementary table 3b. Change-in-estimate for poor SRH to vegetable intake with possible confounding factors (n=6695) .

| Variables removed | Odds ratio | 95% lower limit | 95% upper limit | Change, % |
| --- | --- | --- | --- | --- |
| Adj.All | 1.0492 | 1.0026 | 1.0981 |  |
| **-ethnicity** | **1.0492** | **1.0026** | **1.0980** | **0.0030** |
| **- always lived in this village/town/city** | **1.0494** | **1.0028** | **1.0982** | **0.0203** |
| **-marital status** | **1.0485** | **1.0019** | **1.0972** | **0.0876** |
| **-vigorous fitness/leisure** | **1.0494** | **1.0029** | **1.0982** | **0.0881** |
| -moderate fitness/leisure | 1.0467 | 1.0003 | 1.0952 | 0.2616 |
| -age | 1.0432 | 0.9970 | 1.0914 | 0.3369 |
| -gender | 1.0396 | 0.9938 | 1.0876 | 0.3369 |
| -vigorous level of physical activity | 1.0330 | 0.9877 | 1.0803 | 0.6432 |
| -moderate level of physical activity | 1.0396 | 0.9950 | 1.0863 | 0.6464 |
| -ever schooled | 1.0261 | 0.9824 | 1.0717 | 1.3043 |
| -walk/bike activity | 1.0052 | 0.9629 | 1.0493 | 2.0340 |
| -fruit intake | 0.9794 | 0.9383 | 1.0224 | 2.5657 |

**Supplementary Table 4**

Supplementary table 4a. Change-in-estimate for poor SRH to vigorous level of physical activity with possible confounding factors (n=6770) .

| Variables removed | Odds ratio | 95% lower limit | 95% upper limit | Change, % |
| --- | --- | --- | --- | --- |
| Adj.All | 0.7773 | 0.6439 | 0.9383 |  |
| **-ethnicity** | **0.7770** | **0.6437** | **0.9379** | **0.0435** |
| **-marital status** | **0.7766** | **0.6434** | **0.9375** | **0.0459** |
| - always lived in this village/town/city | 0.7810 | 0.6472 | 0.9426 | 0.5641 |
| -vegetable intake | 0.8014 | 0.6653 | 0.9652 | 2.6051 |
| -gender | 0.7747 | 0.6443 | 0.9314 | 3.3327 |
| -ever schooled | 0.7857 | 0.6540 | 0.9439 | 1.4205 |
| -age | 0.7391 | 0.6163 | 0.8864 | 5.9217 |
| -fruit intake | 0.8144 | 0.6812 | 0.9738 | 10.1896 |

Supplementary table 4b. Change-in-estimate for poor SRH to moderate level of physical activity with possible confounding factors (n=6760).

| Variables removed | Odds ratio | 95% lower limit | 95% upper limit | Change, % |
| --- | --- | --- | --- | --- |
| Adj.All | 0.9668 | 0.8581 | 1.0893 |  |
| **- always lived in this village/town/city** | **0.9668** | **0.8581** | **1.0893** | **0.0014** |
| **-marital status** | **0.9675** | **0.8587** | **1.0901** | **0.0711** |
| **-ethnicity** | **0.9668** | **0.8582** | **1.0892** | **0.0730** |
| -gender | 0.9689 | 0.8601 | 1.0914 | 0.2122 |
| -vegetable intake | 0.9846 | 0.8762 | 1.1064 | 1.6238 |
| -age | 0.9532 | 0.8500 | 1.0690 | 3.1832 |
| -ever schooled | 0.9873 | 0.8813 | 1.1059 | 3.5687 |
| -fruit intake | 1.0565 | 0.9451 | 1.1812 | 7.0175 |

Supplementary table 4c. Change-in-estimate for poor SRH to walk/bike activity with possible confounding factors (n=6755).

| Variables removed | Odds ratio | 95% lower limit | 95% upper limit | Change, % |
| --- | --- | --- | --- | --- |
| Adj.All | 0.5728 | 0.5083 | 0.6455 |  |
| **-ethnicity** | **0.5727** | **0.5082** | **0.6454** | **0.0155** |
| **-marital status** | **0.5725** | **0.5080** | **0.6452** | **0.0360** |
| **-fruit intake** | **0.5721** | **0.5084** | **0.6438** | **0.0728** |
| - always lived in this village/town/city | 0.5703 | 0.5068 | 0.6418 | 0.3071 |
| -ever schooled | 0.5719 | 0.5088 | 0.6428 | 0.2692 |
| -vegetable intake | 0.5742 | 0.5110 | 0.6452 | 0.4046 |
| -gender | 0.5679 | 0.5055 | 0.6379 | 1.1053 |
| -age | 0.5501 | 0.4902 | 0.6173 | 3.1299 |

Supplementary table 4d. Change-in-estimate for poor SRH to vigorous fitness/leisure with possible confounding factors (n=6754).

| Variables removed | Odds ratio | 95% lower limit | 95% upper limit | Change, % |
| --- | --- | --- | --- | --- |
| Adj.All | 0.6567 | 0.4728 | 0.9121 |  |
| **-marital status** | **0.6565** | **0.4727** | **0.9118** | **0.0281** |
| **-ethnicity** | **0.6563** | **0.4726** | **0.9115** | **0.0284** |
| - always lived in this village/town/city | 0.6537 | 0.4708 | 0.9078 | 0.3974 |
| -vegetable intake | 0.6494 | 0.4677 | 0.9017 | 0.6655 |
| -age | 0.6448 | 0.4645 | 0.8949 | 0.7121 |
| -gender | 0.6347 | 0.4574 | 0.8806 | 1.5655 |
| -fruit intake | 0.6067 | 0.4381 | 0.8401 | 4.4115 |
| -ever schooled | 0.5631 | 0.4077 | 0.7778 | 7.1814 |

Supplementary table 4e. Change-in-estimate for poor SRH to moderate fitness/leisure with possible confounding factors (n=6753).

| Variables removed | Odds ratio | 95% lower limit | 95% upper limit | Change, % |
| --- | --- | --- | --- | --- |
| Adj.All | 0.6019 | 0.5031 | 0.7200 |  |
| **-ethnicity** | **0.6016** | **0.5029** | **0.7197** | **0.0434** |
| -age | 0.6022 | 0.5034 | 0.7204 | 0.1011 |
| - always lived in this village/town/city | 0.6017 | 0.5034 | 0.7193 | 0.0813 |
| -gender | 0.6003 | 0.5022 | 0.7175 | 0.2435 |
| -vegetable intake | 0.6022 | 0.5038 | 0.7197 | 0.3198 |
| -marital status | 0.6044 | 0.5057 | 0.7222 | 0.3614 |
| -ever schooled | 0.5689 | 0.4767 | 0.6790 | 5.8638 |
| -fruit intake | 0.5080 | 0.4266 | 0.6049 | 10.7095 |
